# Supplementary material for: Temporal Association of Total Serum Cholesterol and Pancreatic Cancer Incidence
Source: Nutrients. 2022 Nov 21;14(22):4938. doi: 10.3390/nu14224938 (PMC9696583; doi:10.3390/nu14224938)
Supplement: Supplementary file 1 [file nutrients-14-04938-s001.zip › nutrients-2009260-supplementary.pdf]

Table S1. Multivariable odds ratios and 95% confidence intervals of pancreatic cancer incidence by increase of total cholesterol levels (10 mg/dL) during the past 3 years.

Multivariable model	Every 10 mg/dL increase of total cholesterol
Age and sex stratified	0.93 (0.89, 0.98)
Multivariable [1]	0.93 (0.89, 0.98)
Multivariable [1] + statin use	0.94 (0.89, 0.99)
Multivariable [1] + statin use+ cumulative average cholesterol	0.94 (0.89, 0.99)

[1] Multivariable analyses were stratified by age (continuous, years) and sex (men, women); adjusted for smoking status (never smoker, ever smoker), alcohol consumption (yes, no), body mass index (BMI) (<23, 23.0–27.4, ≥27.5 kg/m^2), and regular physical activity (yes, no).

Table S2. Multivariable [1] odds ratios and 95% confidence intervals of pancreatic cancer for patterns of total cholesterol levels among subgroups.

Subgroups	No. of cases/controls	Patterns[2] of fasting blood glucose levels across time intervals (–11 to –4 years, –3 to 0 years)				$P_{interaction}$
		Consistently low cholesterol (L, L)	Recent-onset hypercholesterolemia (L, H)	Recent-resolved hypercholesterolemia (H, L)	Consistent hypercholesterolemia (H, H)	
Sex						
Men	130/390	1 (reference)	0.52 (0.15, 1.81)	2.01 (0.74, 5.42)	1.35 (0.45, 4.03)	0.21
Women	85/255	1 (reference)	0.42 (0.14, 1.26)	1.92 (0.71, 5.22)	0.32 (0.07, 1.43)	
Smoking						
Never smokers	106/338	1 (reference)	0.68 (0.27, 1.73)	2.27(0.91, 5.63)	0.15(0.02, 1.13)	0.18
Ever smokers	109/307	1 (reference)	0.17 (0.02, 1.23)	1.51(0.49, 4.64)	2.05 (0.68, 6.16)	
Alcohol consumption						
Never drinkers	93/273	1 (reference)	0.60 (0.22, 1.64)	1.22 (0.43, 3.51)	0.63 (0.17, 2.30)	0.50
Ever drinkers	122/372	1 (reference)	0.29 (0.07, 1.26)	3.03 (1.14, 8.02)	0.82 (0.26, 2.56)	
BMI						
<23	74/222	1 (reference)	0.51 (0.14, 1.82)	2.69 (0.75, 9.61)	0.83(0.16, 4.19)	0.58
≥23	141/423	1 (reference)	0.42 (0.14, 1.22)	1.53 (0.65, 3.60)	0.66 (0.24, 1.81)	
Physical activity						
Irregular	167/526	1 (reference)	0.53 (0.23, 1.22)	1.86 (0.81, 4.31)	0.48 (0.16, 1.40)	0.34
Regular	48/119	1 (reference)	NA	2.06 (0.56, 7.54)	1.85 (0.38, 9.06)	
Statin use						
Never users	141/458	1 (reference)	0.83 (0.32, 2.01)	1.41 (0.25, 7.97)	1.03 (0.36, 2.93)	0.89
Ever users	74/187	1 (reference)	0.12 (0.02, 0.94)	1.99 (0.92, 4.31)	0.39 (0.08, 1.79)	

Abbreviation: L = lower total serum cholesterol levels (<240 mg/dL), H = higher total serum cholesterol levels (≥240 mg/dL). [1] From unconditional logistic regression adjusted for age (continuous, years), sex (men, women), smoking status (never smoker, ever smoker), alcohol consumption (yes, no), BMI (<23, 23.0–27.4, ≥27.5 kg/m^2), and regular physical activity (yes, no). [2] Temporal patterns of total cholesterol levels (consistently low, recent-onset hypercholesterolemia, recent-resolved hypercholesterolemia, consistent hypercholesterolemia) were mutually adjusted for in multivariable models.

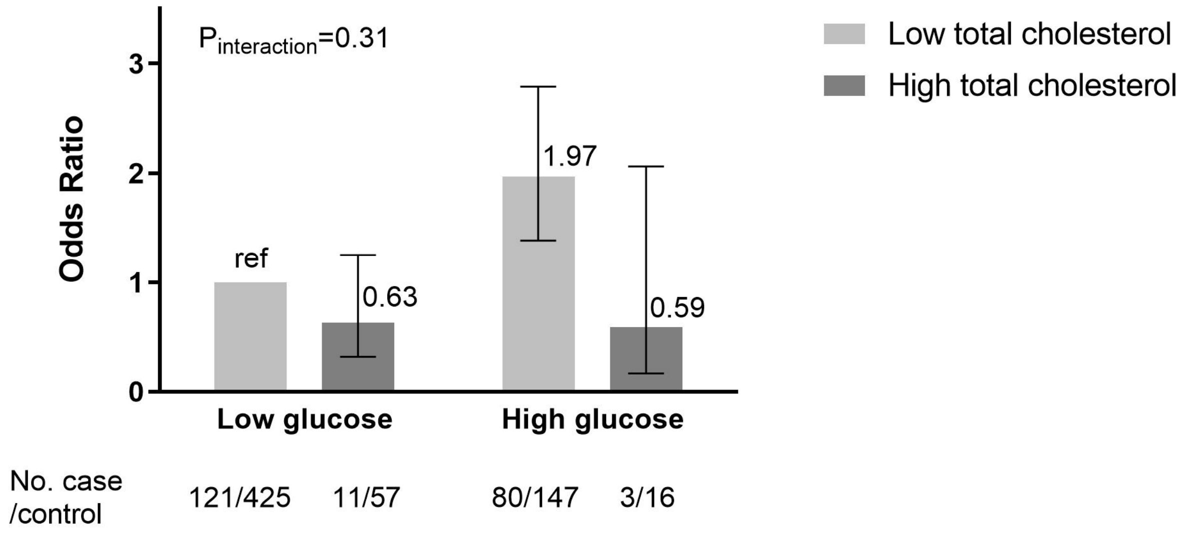

Figure S1. Joint analysis of total serum cholesterol and fasting blood glucose during the recent past 3 years.
